# Molecular Mechanisms of Large-Conductance Ca^**2+**^-Activated Potassium Channel Activation by Ginseng Gintonin

**DOI:** 10.1155/2013/323709

**Published:** 2013-04-04

**Authors:** S. H. Choi, B. H. Lee, S. H. Hwang, H. J. Kim, S. M. Lee, H. C. Kim, H. W. Rhim, S. Y. Nah

**Affiliations:** ^1^Ginsentology Research Laboratory and Department of Physiology, College of Veterinary Medicine and Bio/Molecular Informatics Center, Konkuk University, Seoul 143-701, Republic of Korea; ^2^Neuropsychopharmacology and Toxicology Program, College of Pharmacy, Kangwon National University, Chuncheon 200-701, Republic of Korea; ^3^Life Science Division, KIST, Seoul 130-701, Republic of Korea

## Abstract

Gintonin is a unique lysophosphatidic acid (LPA) receptor ligand found in *Panax ginseng*. Gintonin induces transient [Ca^2+^]_i_ through G protein-coupled LPA receptors. Large-conductance Ca^2+^-activated K^+^ (BK_Ca_) channels are expressed in blood vessels and neurons and play important roles in blood vessel relaxation and attenuation of neuronal excitability. BK_Ca_ channels are activated by transient [Ca^2+^]_i_ and are regulated by various Ca^2+^-dependent kinases. We investigated the molecular mechanisms of BK_Ca_ channel activation by gintonin. BK_Ca_ channels are heterologously expressed in *Xenopus oocytes*. Gintonin treatment induced BK_Ca_ channel activation in oocytes expressing the BK_Ca_ channel **α** subunit in a concentration-dependent manner (EC_50_ = 0.71 ± 0.08 *µ*g/mL). Gintonin-mediated BK_Ca_ channel activation was blocked by a PKC inhibitor, calphostin, and by the calmodulin inhibitor, calmidazolium. Site-directed mutations in BK_Ca_ channels targeting CaM kinase II or PKC phosphorylation sites but not PKA phosphorylation sites attenuated gintonin action. Mutations in the Ca^2+^ bowl and the regulator of K^+^ conductance (RCK) site also blocked gintonin action. These results indicate that gintonin-mediated BK_Ca_ channel activations are achieved through LPA1 receptor-phospholipase C-IP_3_-Ca^2+^-PKC-calmodulin-CaM kinase II pathways and calcium binding to the Ca^2+^ bowl and RCK domain. Gintonin could be a novel contributor against blood vessel constriction and over-excitation of neurons.

## 1. Introduction

Ginseng, the root of *Panax ginseng* C. A. Meyer, has been used as a representative tonic or an adaptogen to promote longevity and to enhance bodily functions against hypertension and as a neuroprotectant for several hundred years in Far East countries like Korea, China, and Japan. Currently, ginseng is one of the most famous and precious herbal medicines consumed around the world [1]. Recently, we isolated and characterized a novel glycolipoprotein, designated as gintonin, from ginseng. Gintonin is a lysophosphatidic-acids- (LPAs-) ginseng major latex-like protein (MLP151) and ginseng ribonuclease-like storage protein complex, in which the lysophosphatidic acids (LPAs) bind to ginseng proteins through hydrophobic interactions, and this is the main principle underlying gintonin action [2–6], whereas most of other LPA receptor ligands are derivatives of LPA or LPA analogs [7]. Gintonin induces transient [Ca^2+^]_i_ through LPA receptor activation via pertussis toxin- (PTX-) sensitive and -insensitive G proteins in animal cells [2–6]. Thus, gintonin-mediated transient [Ca^2+^]_i_ induction via LPA receptors could be further coupled to the regulation of Ca^2+^-dependent enzymes and Ca^2+^-dependent ion channel activities, which play important roles in biological systems.

Large-conductance Ca^2+^-activated K^+^ (BK_Ca_) channels are a family of outward K^+^-selective ion channels activated in response to membrane depolarization. BK_Ca_ channels are activated by intracellular Ca^2+^ elevation and/or Ca^2+^-dependent kinases [8, 9]. BK_Ca_ channels play key roles in neuronal and nonneuronal cell functions. For example, in neuronal cells, BK_Ca_ channels regulate the frequency of firing, action potentials following hyperpolarization, and neurotransmitter release. In vascular smooth muscle cells, BK_Ca_ channels are one of the main ion channels that are involved in vasorelaxation [10, 11].

BK_Ca_ channels are composed of two subunits: the *α* subunit (also called *rSlo*), which forms the channel pore [12], and the *β* subunit [13, 14], which modifies the voltage and calcium sensitivity of the pore-forming *α* subunit [15, 16]. The *α* subunit has a large cytoplasmic C terminus and is responsible for the Ca^2+^-dependent activation of the channel. Furthermore, the cytoplasmic C terminus of the *α* subunit has two domains that are responsible for the Ca^2+^-dependent activation of the channel, namely, the Ca^2+^ bowl and the regulator of K^+^ conductance (RCK) domain [17–21]. The cytoplasmic C terminus of the *α* subunit has amino acid residues that can be phosphorylated by a variety of protein kinases such as CaM kinase II, PKA, and PKC [8, 9]. Accumulating evidence shows that BK_Ca_ channels play key roles in excitable cells and are regulated by diverse Ca^2+^ and Ca^2+^-dependent kinases [10, 11]. Although the signaling pathways of LPA as well as gintonin are well characterized through the biochemical and pharmacological experiments [2–6, 22], relatively little is known about the molecular mechanisms how gintonin-mediated [Ca^2+^]_i_ transient is linked to BK_Ca_ channel regulation.

In the present study, we examined how LPA receptor activation by gintonin may regulate BK_Ca_ channel activity in *Xenopus* oocytes expressing the *α* subunit of BK_Ca_ alone or in *Xenopus* oocytes coexpressing BK_Ca_ channels and other BK_Ca_ channel regulators. We found that treatment of gintonin induces BK_Ca_ channel activation. Gintonin-mediated BK_Ca_ channel activation is achieved through the LPA1 receptor, the phospholipase C-IP_3_-Ca^2+^ pathway, and CaM kinase II phosphorylation of the *α* subunit. We further demonstrated that site-directed mutations of the Ca^2+^ bowl, RCK domain, and CaM kinase II phosphorylation site of channels greatly attenuated gintonin action. We compared the regulatory modes between gintonin and ginsenoside Rg_3_ in BK_Ca_ channel activation. We further discuss how signal coupling of gintonin to the BK_Ca_ channel through the LPA receptor is associated with the beneficial physiological and pharmacological effects of ginseng on blood vessels and the nervous system.

## 2. Materials and Methods

### 2.1. Materials

Gintonin was isolated from *P. ginseng* as described previously [23]. In the present study, we used the crude gintonin fraction, which contains about 9.5% LPAs, the majority being LPA_C18:2_ [2–6]. Ginsenoside Rg_3_ was provided by the AMBO Institute (Seoul, Republic of Korea). The stock solution of ginsenoside Rg_3_ was prepared and used as described previously [24]. M1 muscarinic acetylcholine receptor was purchased from Guthrie Research Institute (Sayre, PA, USA). CaM kinase II gene was kindly provided by OriGene (Rockville, MD, USA). All other reagents were obtained from Sigma-Aldrich (St. Louis, MO, USA).

### 2.2. *In Vitro* Synthesis of cRNA

Recombinant plasmids containing cDNA inserts for M1 muscarinic receptor, *α* subunit (*rSlo*), and constitutively active CaM kinase II were linearized by digestion with appropriate restriction enzymes. The cRNAs from linearized templates were obtained with an *in vitro* transcription kit (mMessage mMachine; Ambion, Austin, TX, USA) using a SP6, T3, or T7 RNA polymerase. The RNA was dissolved in RNase-free water at 1 *μ*g/*μ*L, divided into aliquots, and stored at −70°C.

### 2.3. Preparation of Xenopus Oocytes and Microinjection

*Xenopus laevis* was purchased from Xenopus I (Ann Arbor, MI, USA). Their care and handling were in accordance with the highest standard of institutional guidelines of Konkuk University. For the isolation of oocytes, frogs were anesthetized with an aerated solution of 3-amino benzoic acid ethyl ester followed by the removal of ovarian follicles. The oocytes were subsequently treated with collagenase and then agitated for 2 h in Ca^2+^-free OR2 medium containing 82.5 mM NaCl, 2 mM KCl, 1 mM MgCl_2_, 5 mM HEPES, 2.5 mM sodium pyruvate, 100 units/mL penicillin, and 100 *μ*g/mL streptomycin. Stages V-VI oocytes were collected and stored in ND96 medium (in mM: 96 NaCl, 2 KCl, 1 MgCl_2_, 1.8 CaCl_2_, and 5 HEPES, pH 7.5) supplemented with 50 *μ*g/mL gentamicin. The oocyte-containing solution was maintained at 18°C with continuous gentle shaking and was renewed daily. Electrophysiological experiments were performed within 5-6 days of oocyte isolation, with gintonin or ginsenoside applied to the bath. For BK_Ca_ channel experiments, BK_Ca_ channel-encoding cRNAs (40 nl) were injected into the animal or vegetal pole of each oocyte one day after isolation, using a 10-*μ*L microdispenser (VWR Scientific, San Francisco, CA, USA) fitted with a tapered glass pipette tip (diameter, 15–20 *μ*m) [25].

### 2.4. Site-Directed Mutagenesis of the BK_**Ca**_ *α* and *In Vitro* Transcription of BK_**Ca**_ Channel cDNAs

**Figure 1:**
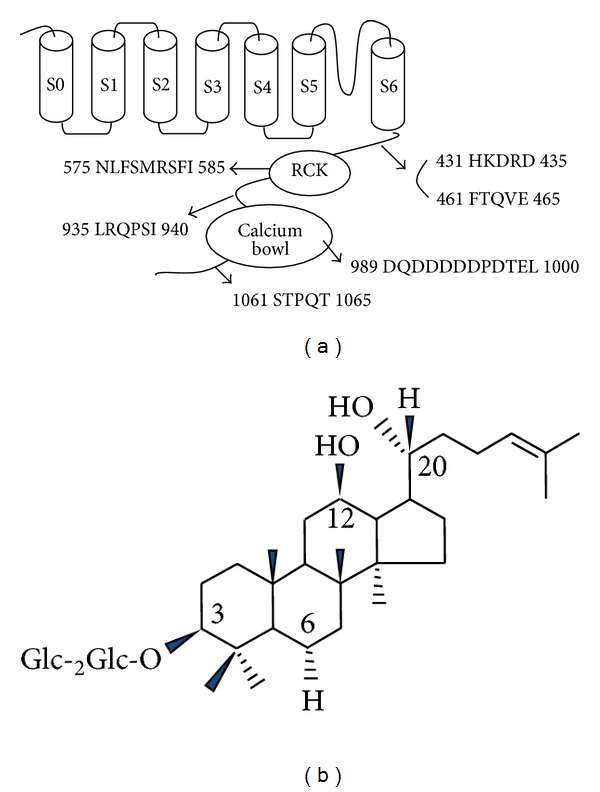
The primary amino acid sequence of the mutated BK_Ca_ channel *α* subunit and the chemical structure of ginsenoside Rg_3_. (a) The brief sequence alignment of the BK_Ca_ channel and the mutated amino acid residues in the CaM kinase II phosphorylation sites (462, 512), PKA (939), PKC (1061), or Ca^2+^ binding sites of the Ca^2+^ bowl (989, 991, 992, 993, 994, 995) and RCK domains (433, 579). (b) The chemical structure of ginsenoside Rg_3_. Glc: glucose.

Single amino acid substitutions of the BK_Ca_ channel (Figure 1(a)) were made using a QuikChange XL Site-Directed Mutagenesis Kit (Stratagene, La Jolla, CA, USA), along with Pfu DNA polymerase and sense and antisense primers encoding the desired mutations. Overlap extension of the target domain by sequential polymerase chain reaction (PCR) was carried out according to the manufacturer's protocol. The final PCR products were transformed into *E. coli* strain DH5*α*, screened by PCR, and confirmed by sequencing of the target regions. The mutant DNA constructs were linearized at their 3′ ends by digestion with *Not*I, and run-off transcripts were prepared using the methylated cap analog, m7G(5′)ppp(5′)G. The cRNAs were prepared using an mMessage mMachine transcription kit (Ambion, Austin, TX, USA) with T7 RNA polymerase. The absence of degraded RNA was confirmed by denaturing agarose gel electrophoresis followed by ethidium bromide staining. Similarly, recombinant plasmids containing rat BK_Ca_ channel cDNA inserts were linearized by digestion with the appropriate restriction enzymes, and cRNAs were obtained using the mMessage mMachine *in vitro* transcription kit (Ambion, Austin, TX, USA) with SP6 RNA polymerase or T7 polymerase. The final cRNA products were resuspended at a concentration of 1 *μ*g/*μ*L in RNase-free water and stored at −80°C [25].

### 2.5. Data Recording

 Data recording for BK_Ca_ channel currents was performed, as described by Liu et al. [26], to study the detailed downstreams of gintonin-mediated signaling transduction pathways. Therefore, a custom-made Plexiglas net chamber was used for two-electrode voltage-clamp recordings, as previously reported [25]. The oocytes were impaled with two microelectrodes filled with 3 M KCl (0.2–0.7 MΩ), and electrophysiological experiments were carried out at room temperature using an Oocyte Clamp (OC-725C, Warner Instruments, Hamsden, CT, USA). Stimulation and data acquisition were controlled with a pClamp 8 (Axon Instruments, Union City, CA, USA). For most electrophysiological experiments, oocytes were perfused initially with a Cl^−^, and Ca^2+^-free solution (in mM: 96 NaOH, 2 KOH, 8 Mg-gluconate, 5 HEPES, and 5 EGTA, pH 7.4 with methanesulfonic acid) in the presence of a Cl^−^ channel blocker (500 *μ*M anthracene-9-carboxylic acid) [26] to inhibit endogenous Cl^−^ channels. The oocytes were then clamped at a holding potential of −80 mV; membrane potential was depolarized to +40 mV for 400 ms at 10-s intervals, and currents were recorded as indicated.

### 2.6. Data Analysis

To obtain the concentration-response curve of the effect of gintonin or ginsenoside Rg_3_ on the K^+^ currents from the BK_Ca_ channel, the peak amplitudes at different concentrations of gintonin were plotted. The current activation or enhancement evoked by drug treatment was analyzed after the subtraction of currents elicited by H_2_O injection. Origin software (Origin, Northampton, MA, USA) was used to plot the Hill equation: *y*/*y*_max⁡_ = [*A*]^nH^/([*A*]^nH^ + [EC_50_]^nH^), where *y* represented the peak current at a given concentration of gintonin, *y*_max⁡_ was the maximal peak current, EC_50_ was the concentration of gintonin producing a half-maximal effect, [*A*] was the concentration of gintonin, and nH was the Hill coefficient. All values are presented as mean ± S.E.M. The significance of differences between mean control and treatment values was determined using Student's *t*-test, where *P* < 0.05 was considered statistically significant.

## 3. Results

### 3.1. Gintonin Induces BK_**Ca**_ Channel Activation in a Concentration-Dependent and Voltage-Dependent Manner in Xenopus Oocytes Expressing BK_**Ca**_ Channels

**Figure 2:**
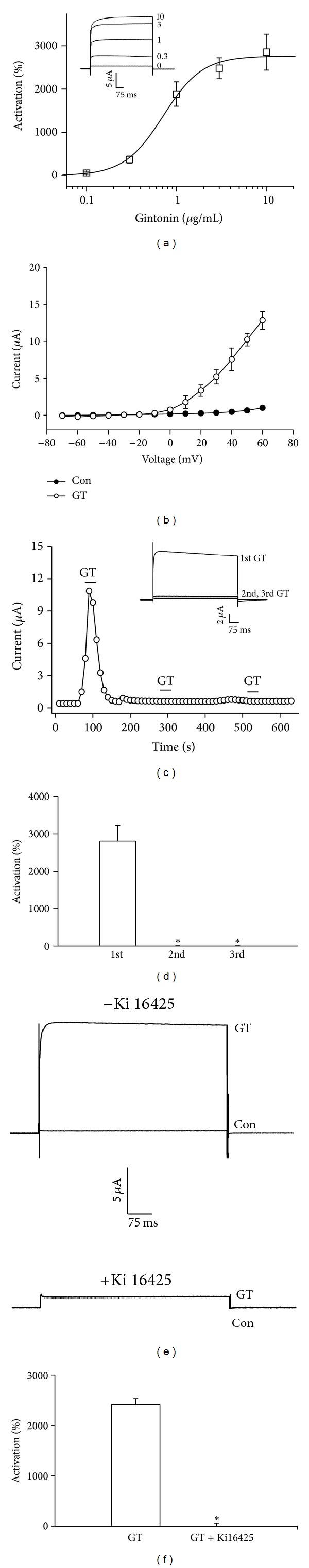
Effects of gintonin on BK_Ca_ channel activity. (a) Gintonin concentration-response curve (mean ± S.E.M; *n* = 13–15 oocytes each). *Inset*, the representative traces of gintonin-mediated BK_Ca_ channel activation at various concentrations. (b) Effects of gintonin (10 *μ*g/mL) on the current-voltage (*I*-*V*) relationship of the wild-type BK_Ca_ channel (mean ± S.E.M; *n* = 13–15 oocytes each). (c) Time-current relationship plotted against time before and after repeated applications of gintonin (10 *μ*g/mL) for 30 s in oocytes expressing the BK_Ca_ channel. *Inset*, the representative peak outward current amplitude at +40 mV from a holding potential of −80 mV was measured in the absence or presence of gintonin. (d) Summary histograms show that repeated application of gintonin induces the desensitization of gintonin-mediated BK_Ca_ channel activation (*P < 0.001, compared to 1st gintonin treatment). (e) The representative traces on blockage of gintonin-mediated BK_Ca_ channel activation by the LPA1/3 receptor antagonist, Ki16425. (f) Summary histograms show that gintonin-mediated activation of the BK_Ca_ channel is blocked by the LPA1/3 receptor antagonist, Ki16425 (mean ± S.E.M; *n* = 12–14 each) (*P < 0.001, compared to gintonin alone).

In the present study, we first examined the effect of gintonin on BK_Ca_ channel activity in *Xenopus* oocytes expressing BK_Ca_ channel *α* (*rSlo*) subunits. Application of gintonin to oocytes injected with *α* subunit cRNAs resulted in the activation of the BK_Ca_ channel as monitored with a clamp step to +60 from −80 mV holding potential (*n* = 7; current examined at 20-s intervals). The mean current activation by gintonin (10 *μ*g/mL) was 2855.1 ± 415.0% (Figure 2(a)), whereas gintonin had no effect on the control oocytes that were not injected with cRNA encoding the BK_Ca_ *α* subunit gene (data not shown). Gintonin-induced BK_Ca_ channel activation occurred in a concentration-dependent manner (Figure 2(a)). The EC_50_ was observed to be 0.71 ± 0.08 *μ*g/mL. Charybdotoxin and iberiotoxin, highly specific inhibitors of maxi-K channels [27, 28], greatly attenuated BK_Ca_ channel activation induced by gintonin (data not shown), indicating that BK_Ca_ channels are functional [29]. BK_Ca_ channel activation by gintonin was observed over the entire voltage range examined from 0 mV. Thus, gintonin-induced BK_Ca_ channel activation occurs in a voltage-dependent manner since marked activations at more positive potentials were observed, as shown in a current-voltage relationship (Figure 2(b)).

### 3.2. Gintonin-Induced BK_**Ca**_ Channel Activation Is Rapidly Desensitized following Repeated Application of Gintonin and Is Blocked by a LPA1/3 Receptor Antagonist

We next examined the changes in gintonin-induced BK_Ca_ channel activation following repeated application of gintonin. As shown in Figures 2(c) and 2(d), an initial treatment of gintonin induced a marked activation of BK_Ca_ channels. Oocytes stimulated with gintonin were then washed with recording buffer for 3 min until the basal current was recovered and subsequently restimulated with gintonin. We observed that secondary and tertiary BK_Ca_ channel current responses to gintonin retreatment were dramatically diminished. The magnitudes of the BK_Ca_ current were 2700 ± 121.6, 55.3 ± 33.1, and 26 ± 10.6%, respectively, of the initial, secondary, or tertiary responses of gintonin treatment (*n* = 15, oocytes each; from three different batches of donors) (Figure 2(d)). We next examined the effect of an LPA1/3 receptor antagonist, Ki16425, on gintonin-induced BK_Ca_ channel activation. In the presence of Ki16425, gintonin-mediated BK_Ca_ channel activation was abolished, from 2410 ± 115.56 to 10.64 ± 50.54% (Figures 2(e) and 2(f)). This result indicates that gintonin-mediated BK_Ca_ channel activation was achieved through activation of the LPA receptor in *Xenopus* oocytes, which endogenously express LPA1 receptors [30].

### 3.3. The Signal Transduction Pathway of Gintonin-Mediated BK_**Ca**_ Channel Activation

**Figure 3:**
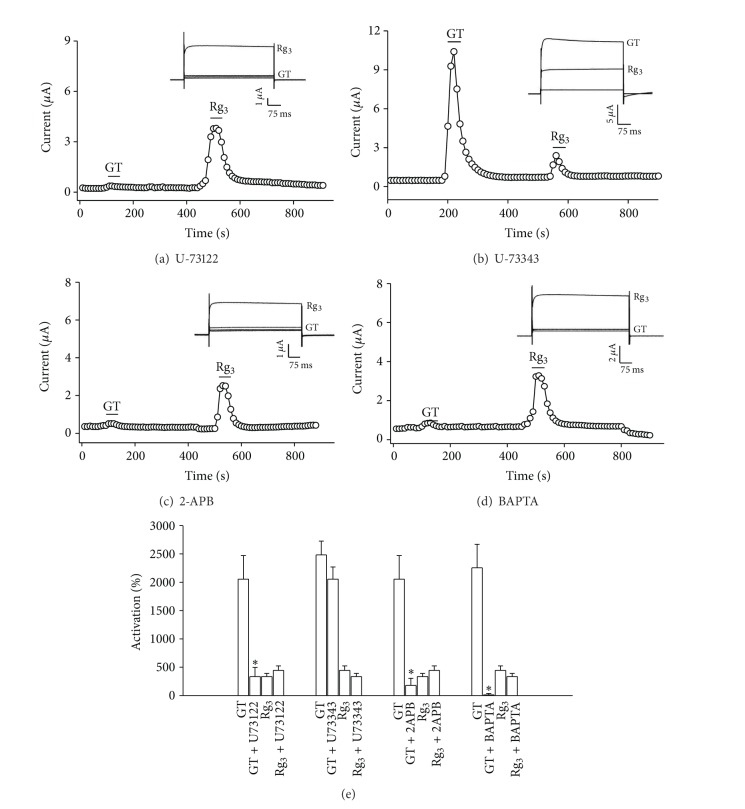
The signal transduction pathways in gintonin-mediated BK_Ca_ channel activation. ((a) and (b)) Time-current relationship following application of gintonin (10 *μ*g/mL) or ginsenoside Rg_3_ (100 *μ*M) for 30 s in the presence of U73122, an active PLC inhibitor, or U73343, an inactive PLC inhibitor, in oocytes expressing BK_Ca_ channels. *Inset*, the representative peak outward current amplitude at +40 mV from a holding potential of −80 mV was measured in the presence of gintonin or ginsenoside Rg_3_. The active or inactive PLC inhibitor was pretreated for 5 min before gintonin or ginsenoside Rg_3_ application. ((c) and (d)) Time-current relationship after application of gintonin (10 *μ*g/mL) or ginsenoside Rg_3_ (100 *μ*M) for 30 s in the presence of 2-APB, an IP_3_ receptor antagonist, or BAPTA, an intracellular Ca^2+^ chelator, in oocytes expressing BK_Ca_ channels. *Inset*, the representative peak outward current amplitude at +40 mV from a holding potential of −80 mV was measured in the presence of gintonin or ginsenoside Rg_3_. The application of 2-APB or BAPTA preceded the gintonin application by 2 h. (e) Summary histograms show the peak outward BK_Ca_ channel currents (mean ± S.E.M; *n* = 13-14 oocytes each) recorded in oocytes expressing the BK_Ca_ channel in the absence or presence of the indicated agents. (*P < 0.001, compared to gintonin alone).

We next examined the signal transduction pathways involved in gintonin-mediated BK_Ca_ channel activation. We first examined the involvement of phospholipase C (PLC) in gintonin-mediated BK_Ca_ channel activation. To test this possibility, the effects of the active PLC inhibitor U-73122 and its inactive analogue U-73343 were examined on gintonin action [31]. Bath application of U-73122 significantly suppressed gintonin action, whereas the current in the presence of U-73343 was not affected (Figures 3(a) and 3(b)). These results indicate that gintonin-mediated BK_Ca_ channel activation requires PLC activation.

To see if the IP_3_ receptor was involved in gintonin action, oocytes were stimulated with gintonin in the presence of 2-APB, an IP_3_ receptor antagonist. We observed that 2-APB treatment also greatly attenuated the effect of gintonin on BK_Ca_ channel activation (Figure 3(c)). These observations suggest that gintonin first induces an activation of the IP_3_ receptor to mobilize intracellular Ca^2+^, and then mobilized [Ca^2+^]_i_ is coupled to BK_Ca_ channel activation. We have demonstrated in a previous report that gintonin-mediated activation of Ca^2+^-activated Cl^−^ channels is dependent on cytosolic Ca^2+^ [23]; thus, we examined whether the effect of gintonin on BK_Ca_ channel activation was also dependent on cytosolic Ca^2+^. To this end, we first treated oocytes with BAPTA-AM, a membrane permeable Ca^2+^ chelator, to chelate free cytosolic Ca^2+^ and examined gintonin effects. We found that BAPTA-AM completely abolished gintonin action on BK_Ca_ channel activation (Figure 3(d)), indicating that gintonin-mediated BK_Ca_ channel activation is also achieved through mobilization of intracellular Ca^2+^ from endoplasmic reticulum. However, while ginsenoside Rg_3_ (100 *μ*M) also enhanced BK_Ca_ channel currents, as shown previously [24], gintonin exhibited a greater (3-4-fold) activation of the BK_Ca_ channel than ginsenoside Rg_3_. In addition, the enhancing effects of ginsenoside Rg_3_ on BK_Ca_ channel currents were not sensitive to the PLC inhibitor, the 2-APB antagonist, and BAPTA (Figure 3(e)), indicating that the regulatory mode on BK_Ca_ channel activity by gintonin differs from that of ginsenoside Rg_3_.

### 3.4. Involvement of PKC but Not PKA in Gintonin-Mediated BK_**Ca**_ Channel Activation

**Figure 4:**
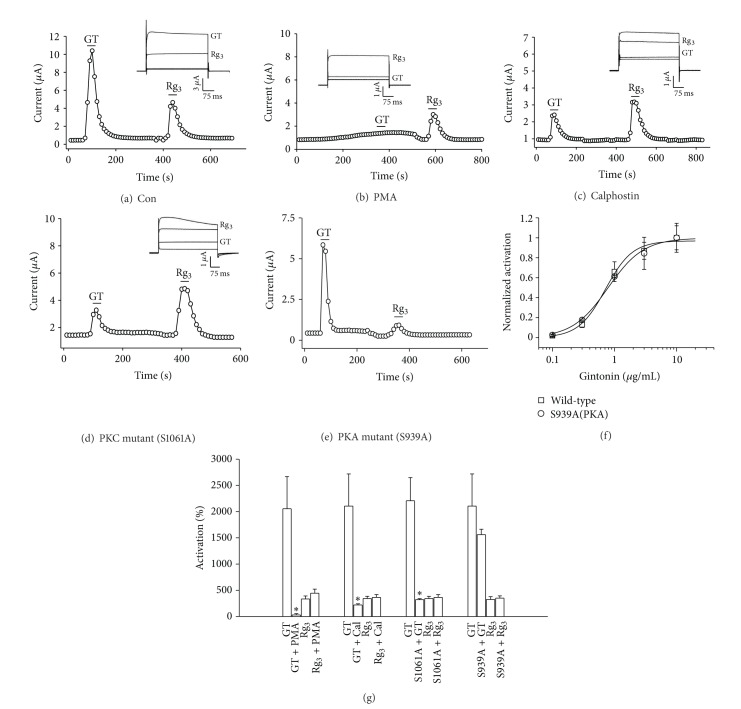
Involvement of PKC but not PKA in gintonin-mediated BK_Ca_ channel activation. (a) Gintonin or ginsenoside Rg_3_ induces activation of BK_Ca_ channels in oocytes. ((b)–(d)) Time-current relationships show the effects of gintonin or ginsenoside Rg_3_ in the pretreatment of PMA (1 *μ*M) for 10 min, calphostin (Cal 1.5 *μ*M) for 10 min, or the mutation of the PKC phosphorylation site (S1061A). Peak outward currents were recorded during bath application of gintonin (10 *μ*g/mL). *Insets*, the representative gintonin-mediated or ginsenoside Rg_3_-mediated peak outward current amplitude at +40 mV from a holding potential of −80 mV was measured in the presence of PMA, calphostin, or mutant BK_Ca_ channels. (e) Time-current relationships following the application of gintonin (10 *μ*g/mL) or ginsenoside Rg_3_ (100 *μ*M) for 30 s in oocytes expressing S939A mutant BK_Ca_ channels. (f) Concentration dependency of wild-type and S939A mutant BK_Ca_ channels on gintonin-mediated BK_Ca_ channel activation. (g) Summary histograms show that peak outward BK_Ca_ channel currents (mean ± S.E.M; *n* = 11-12 oocytes each) recorded in oocytes expressing the BK_Ca_ channel in the absence or presence of the indicated agents or mutation (*P < 0.001, compared to gintonin alone).

The previous reports have shown that the activation of the PLC pathway also produces lipid-soluble 1,2-diacylglycerol (DAG), an endogenous protein kinase C (PKC) activator. Activation of PKC by treatment with PMA, a DAG analogue, causes receptor phosphorylation and receptor uncoupling from PLC-mediated inositol phospholipid metabolism and results in a loss of Ca^2+^-activated Cl^−^ channel activation by 5-HT or muscarinic acetylcholine receptor agonist stimulations in *Xenopus* oocytes [4, 5, 12, 32]. Similarly, in the present study we also first examined the effects of the PKC activator PMA on the gintonin-mediated BK_Ca_ channel activation. As shown in Figure 4(b), we found that treatment with PMA induced a loss of gintonin-mediated BK_Ca_ channel activation. To confirm PKC involvement in gintonin-mediated BK_Ca_ channel activation, we next examined the effect of PKC inhibitor, calphostin, with gintonin action and found that calphostin also prevented gintonin-mediated BK_Ca_ channel activation (Figure 4(c)), indicating that gintonin-mediated BK_Ca_ channel activation is achieved via PKC activation through LPA receptor. We also tested whether mutation of the PKC phosphorylation site affects gintonin-mediated BK_Ca_ channel activation. As shown in Figure 4(d), mutation of Ser1061 to S1061A significantly attenuated gintonin-mediated BK_Ca_ channel activation [33]. Similarly, we examined whether mutation of the PKA phosphorylation site affects gintonin-mediated BK_Ca_ channel activation. Interestingly, we found that mutation of the PKA phosphorylation site did not affect gintonin-mediated BK_Ca_ channel activation (Figures 4(e) and 4(f)). Thus, these results indicate that channel protein phosphorylation by PKC, but not PKA, is involved in gintonin-mediated BK_Ca_ channel activation. However, the enhancing effects of ginsenoside Rg_3_ on BK_Ca_ channel currents were not affected by PMA, calphostin, and mutant BK_Ca_ channels at the PKC phosphorylation site (Figure 4(g)). These results collectively indicate that the gintonin-mediated but not ginsenoside Rg_3_-mediated BK_Ca_ channel activation involves PKC activation.

### 3.5. Involvement of Calmodulin and Calcium-/Calmodulin-Dependent Kinase II (CaM Kinase II) in Gintonin-Mediated Activation of Channels

**Figure 5:**
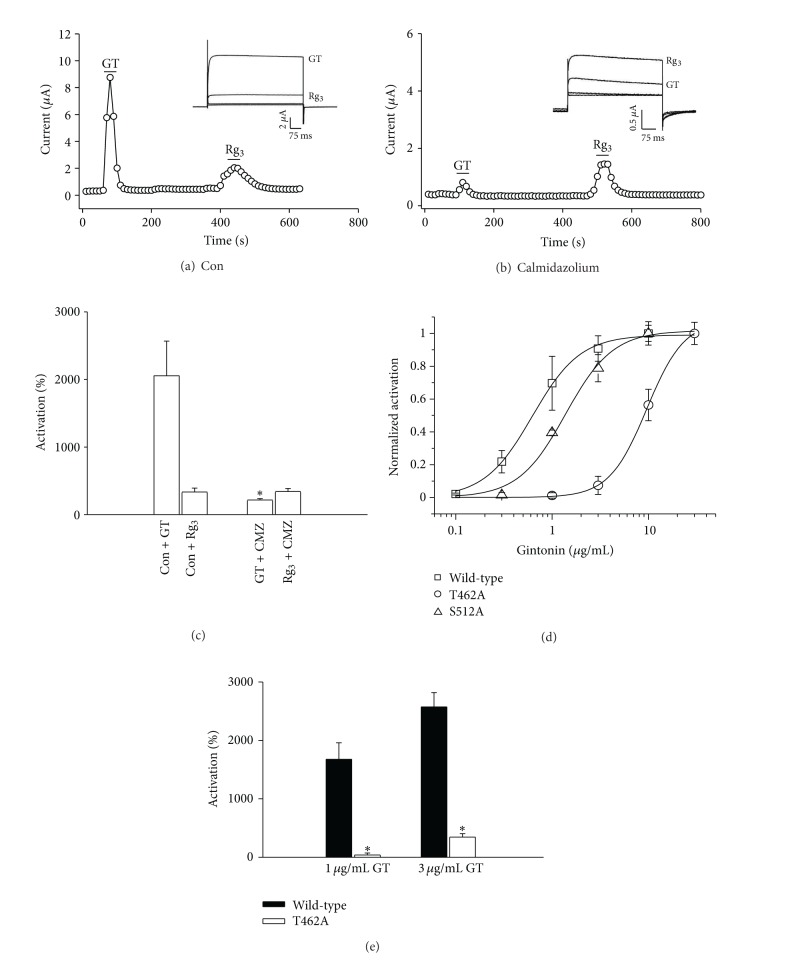
Involvement of calmodulin and CaM kinase II in gintonin-mediated BK_Ca_ channel activation. ((a) and (b)) Oocytes expressing the BK_Ca_ channel were incubated in the absence (a) or presence (b) of calmidazolium (1.5 *μ*M) for 10 min. *Insets*, the representative gintonin-mediated or ginsenoside Rg_3_-mediated peak outward current amplitude at +40 mV from a holding potential of −80 mV was measured in the absence or presence of calmidazolium. (c) Summary histograms show peak outward BK_Ca_ channel currents recorded in oocytes expressing the BK_Ca_ channel in the absence or presence of the calmidazolium (CMZ) (mean ± S.E.M; *n* = 13-14 oocytes each; *P < 0.001, compared to gintonin alone). (d) The oocytes expressing mutant BK_Ca_ channel at the CaM kinase II phosphorylation site (T462A or S521A) were treated with gintonin by bathing application for 60 s. Mutation of CaM kinase II phosphorylation sites resulted in a rightward shift of the gintonin concentration-response curve (mean ± S.E.M; *n* = 10–12 oocytes each). (e) Summary histograms show that the gintonin-mediated peak outward BK_Ca_ channel currents recorded in oocytes expressing mutant BK_Ca_ channel at the CaM kinase II phosphorylation site (T462A or S521A) were significantly attenuated (mean ± S.E.M; *n* = 10–12 oocytes each; *P < 0.001, compared to wild-type).

Since calmodulin and CaM kinase II have been reported to be involved in the regulation of BK_Ca_ channel activation [8], we determined whether calmodulin and CaM kinase II are involved in gintonin-mediated BK_Ca_ channel activation. To this end, we first examined gintonin-mediated BK_Ca_ channel activation following treatment with the calmodulin antagonist, calmidazolium. As shown in Figure 5(b), calmidazolium treatment significantly attenuated gintonin-mediated BK_Ca_ channel activation, indicating that calmodulin is involved in gintonin-mediated BK_Ca_ channel activation. In contrast, the enhancing effects of ginsenoside Rg_3_ on BK_Ca_ channel currents were not affected by calmidazolium (Figure 5(c)). Since calmodulin is closely related with CaM Kinase II activation, which is known to regulate BK_Ca_ channel activity [34, 35], we next examined if gintonin-mediated BK_Ca_ channel activation is achieved through CaM kinase II. To this end, we constructed two different kinds of mutant BK_Ca_ channels at CaM kinase II phosphorylation sites, T462 and S512, by replacing these residues with alanine (T462A and S512A) [26]. As shown in Figures 5(d) and 5(e), the concentration-response curve shifted rightward, indicating that gintonin-mediated BK_Ca_ channel activation was greatly attenuated in mutant channels compared to wild-type channels. Thus, the EC_50_ was 0.62 ± 0.04, 9.61 ± 0.15, and 1.38 ± 0.25 *μ*g/mL in wild-type and T462A and S521A mutants, respectively. Interestingly, gintonin action on BK_Ca_ channel activation was more strongly inhibited in T462A rather than S512A mutants. These results indicate that gintonin induces CaM kinase II activation and links to BK_Ca_ channel activation through BK_Ca_ channel phosphorylation at T462 and S512.

### 3.6. Involvement of the Ca^2+^-Binding Domain (Ca^2+^ Bowl) and RCK Domain in Gintonin-Mediated BK_**Ca**_ Channel Activation

BK_Ca_ channels have unique structures called the Ca^2+^ bowl and the RCK domain. These two domains play important roles in Ca^2+^-dependent regulation of BK_Ca_ channels [36, 37]. To confirm the involvement of the Ca^2+^ bowl and RCK domains in gintonin-mediated BK_Ca_ channel activation, we mutated residues at these domains since C-terminus mutations have been shown to affect Ca^2+^-mediated regulation of BK_Ca_ channel activity [17, 38]. To this end, we constructed six different mutant BK_Ca_ channels in Ca^2+^-bowl residues, D989, D991, D992, D993, D994, and D995, by replacing these residues with alanine (D989A, D991A, D992A, D993A, D994A, and D995A) [39]. Moreover, we constructed two different kinds of mutant BK_Ca_ channels in RCK domain residues such as D433 and M579 by replacing these residues with alanine and isoleucine (D433A and M579I) [21].

**Figure 6:**
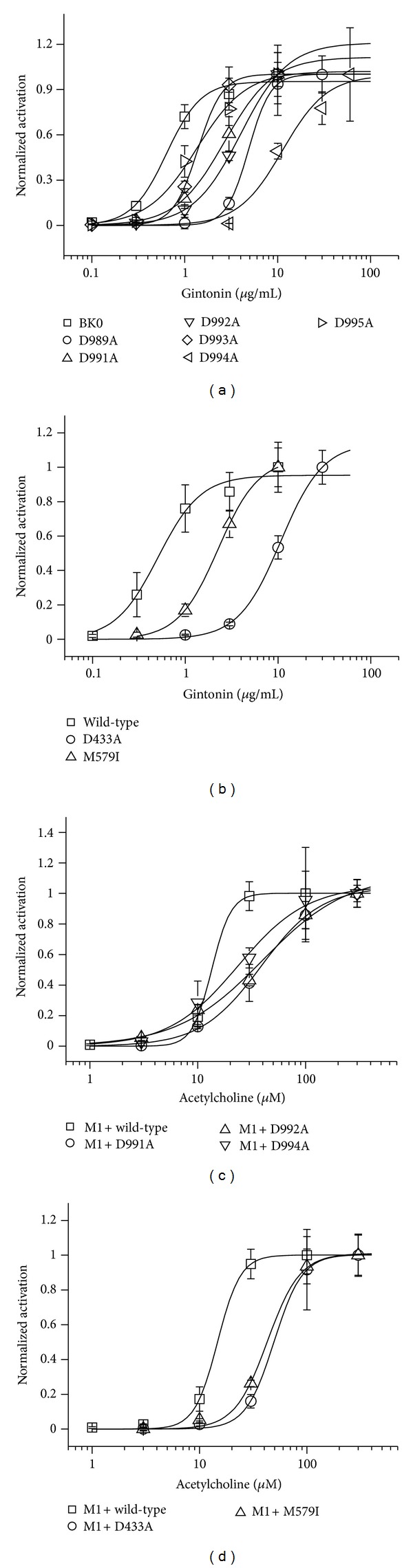
Involvement of the Ca^2+^ bowl and RCK domain in gintonin-mediated BK_Ca_ channel activation. (a) The oocytes expressing wild-type or various mutant BK_Ca_ channels at the Ca^2+^ bowl were treated by bath application of gintonin (10 *μ*g/mL) for 60 s, and peak outward currents were recorded. Mutations of the Ca^2+^ bowl caused a rightward shift of the gintonin concentration-response curve. (b) The oocytes expressing wild-type or RCK domain mutant BK_Ca_ channels, D433A or M579I, were treated with gintonin by bathing application for 60 s. Mutation of the RCK domain also caused a rightward shift of the gintonin concentration-response curve. (c) The oocytes coexpressing M1 muscarinic receptor or various mutant BK_Ca_ channels with M1 muscarinic receptor were treated by bath application of acetylcholine (100 *μ*M), and peak outward currents were recorded. Mutations of the calcium bowl caused a rightward shift of the acetylcholine concentration-response curve. (d) Oocytes coexpressing wild-type with M1 muscarinic receptor or mutant BK_Ca_ channels at the RCK domain such as D433A or M579I with M1 muscarinic receptor were treated with gintonin by bathing application for 60 s. Mutations in the RCK domain caused a rightward shift of the gintonin concentration-response curve (mean ± S.E.M; *n* = 13-14 oocytes each).

We then examined the effects of gintonin on the activity of these mutant channels. In concentration-response curves, the stimulatory effects of gintonin on BK_Ca_ channel activity were observed to be greatly attenuated in oocytes expressing the mutants compared to wild-type channels in the order of D994A > D989A > D922A > D991 > D993A (Figure 6(a)). The EC_50 _values were 0.64 ± 0.08, 1.00 ± 0.01, 2.70 ± 0.06, 4.01 ± 0.06, 1.38 ± 0.03, 11.31 ± 4.62, and 1.35 ± 0.22 *μ*g/mL in wild-type, D989A, D991A, D992A, D993A, D994A, and D995A, respectively. Interestingly, gintonin action on BK_Ca_ channel activation was more strongly inhibited in D994A rather than in other Ca^2+^ bowl mutants. We also examined the effects of gintonin on RCK domain mutant channels. As shown in Figure 6(b), gintonin-mediated BK_Ca_ channel activation was greatly attenuated in oocytes expressing the mutant channels D433A and M579I compared to wild-type channels. The representative concentration-response curves are also shown in Figure 6(b). The EC_50_ values were 0.51 ± 0.07, 10.71 ± 0.60, and 2.26 ± 0.06 *μ*g/mL in wild-type, D433A, and M579I mutants, respectively. Interestingly, gintonin action on BK_Ca_ channel activation was more strongly inhibited in D433A rather than M579I RCK domain mutants. As a positive control, we injected cRNA encoding M1 muscarinic acetylcholine receptor (mAChR) into the oocytes, which are reported to induce transient [Ca^2+^]_i_ via the G*α*_q/11_-PLC-IP_3_ pathway [40]. As shown in Figure 6(c), in Ca^2+^ bowl mutants such as D991A, D992A, and D994A mutants, treatment of acetylcholine caused a right shift in the concentration-response curves. In RCK domain mutants, such as D433S and M579I mutants, treatment of acetylcholine also caused a right shift of the concentration-response curves (Figure 6(d)). These results indicate that the released Ca^2+^ induced by gintonin or acetylcholine treatment binds to the Ca^2+^ bowl and RCK domain and induces BK_Ca_ channel activation. 

### 3.7. Dual Mutations of the Ca^2+^ Bowl or RCK Domain with BK_**Ca**_ Channel Phosphorylation Sites Further Attenuate Gintonin-Mediated BK_**Ca**_ Channel Activation

**Figure 7:**
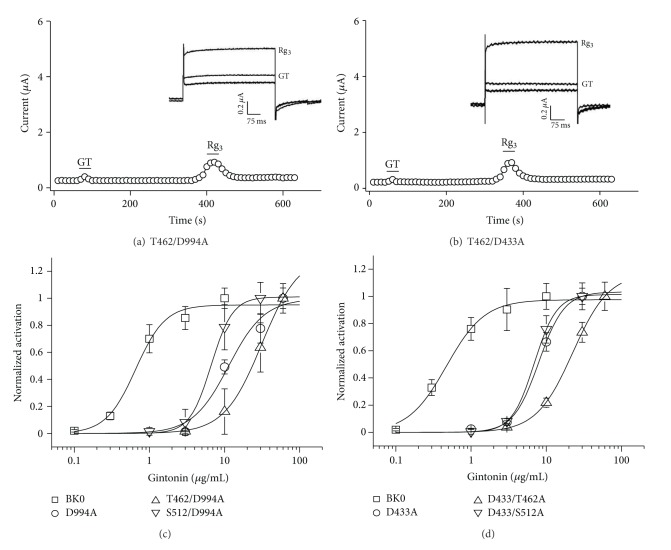
Double mutations of CaM kinase II and Ca^2+^ bowl or RCK domain further attenuate gintonin-mediated BK_Ca_ channel activation. (a) Time-current relationship after application of gintonin (10 *μ*g/mL) or ginsenoside Rg_3_ (100 *μ*M) for 60 s in oocytes coexpressing BK_Ca_ channels and CaM kinase II + Ca^2+^ bowl mutants. *Insets*, the representative peak outward current amplitude at +40 mV from a holding potential of −80 mV was measured in the presence of gintonin or ginsenoside Rg_3_. (b) Time-current relationship after application of gintonin (10 *μ*g/mL) or ginsenoside Rg_3_ (100 *μ*M) for 60 s in oocytes coexpressing BK_Ca_ channels and CaM kinase II + RCK domain mutants. *Inset*, the representative peak outward current amplitude at +40 mV from a holding potential of −80 mV was measured in the presence of gintonin or ginsenoside Rg_3_. (c) Coexpression of BK_Ca_ channels with CaM kinase II + Ca^2+^ bowl mutants caused a further rightward shift of the gintonin concentration-response curve. (d) Coexpression of BK_Ca_ channels with CaM kinase II + RCK domain mutants caused a further rightward shift of the gintonin concentration-response curve (mean ± S.E.M; *n* = 13 oocytes each).

We further examined whether dual mutations of amino acid residues in the Ca^2+^ bowl or the RCK domain and in CaM kinase II phosphorylation sites in the BK_Ca_ channel further affect gintonin-mediated BK_Ca_ channel activation. As shown in Figure 7(a), double mutations of CaM kinase II and the Ca^2+^ bowl (T462/F994A) further attenuated the gintonin-mediated BK_Ca_ channel activation with a concomitant right shift of the concentration-response curves (Figure 7(c)). The EC_50_ was 31.23 ± 1.20 *μ*g/mL. Additionally, double mutations of CaM kinase II and the RCK domain further attenuated the gintonin-mediated BK_Ca_ channel activation (Figure 7(d)). The EC_50_ was 22.5 ± 0.80 *μ*g/mL, again confirming that gintonin-mediated BK_Ca_ channel activation includes Ca^2+^-mediated CaM kinase II activation and Ca^2+^ binding to the Ca^2+^ bowl and RCK region. However, the enhancing effects of ginsenoside Rg_3_ on BK_Ca_ channel currents were not affected by the double mutations of CaM kinase II and the Ca^2+^ bowl or by mutations in CaM kinase II and the RCK domain (Figures 7(a) and 7(b)).

## 4. Discussion

BK_Ca_ channels exist in excitable cells such as neurons and vascular smooth muscle cells. Their main roles are to induce repolarization following depolarization or to restore the resting membrane potential of neurons and vascular smooth muscles. Thus, the physiological functions of BK_Ca_ channels are to regulate synaptic transmission in the nervous system and to relax the blood vessels. The activation of BK_Ca_ channels is closely linked to transient [Ca^2+^]_i_ induction by voltage-gated Ca^2+^ channel activation after depolarization since BK_Ca_ channels colocalize with Ca^2+^ channels [41, 42]. The cytoplasmic C terminus of the BK_Ca_ channel *α* subunit contains two main Ca^2+^ binding sites, that is, the Ca^2+^ bowl and a high Ca^2+^ affinity RCK domain [37]. In addition, various kinases also regulate BK_Ca_ channel activities through the phosphorylation of BK_Ca_ channel proteins [43, 44].

The present study was performed to elucidate the molecular mechanisms coupling gintonin to BK_Ca_ channel activation by using a *Xenopus* oocyte gene expression system. Our results revealed four major findings. Firstly, we observed that gintonin treatment induced BK_Ca_ channel activation in a concentration- and voltage-dependent manner via LPA receptor activation but the repeated treatment of gintonin induced a rapid desensitization. Secondly, the presence of a PLC inhibitor, an IP_3_ receptor, antagonist, an intracellular Ca^2+^ chelator, or a PKC inhibitor greatly attenuated the action, of gintonin. Thirdly, treatment with a calmodulin inhibitor attenuated gintonin action and mutations of PKC and CaM kinase II phosphorylation sites, but not by PKA phosphorylation sites, on the BK_Ca_ channel greatly attenuated gintonin action. Fourthly, mutations of amino acid residues in the Ca^2+^ bowl and RCK domains greatly attenuated gintonin-mediated enhancement of BK_Ca_ channel currents. Thus, since BK_Ca_ channels play an important role in presynaptic nerve terminals and blood vessel smooth muscle cells, the findings in the present study show the possibility that gintonin may be a novel BK_Ca_ channel regulator in the nervous and vascular systems via the PLC-IP_3_-Ca^2+^ and Ca^2+^-PKC-CaM kinase II signal transduction pathways.

Interestingly, although gintonin- and acetylcholine-mediated BK_Ca_ channel activations are attenuated by site-directed mutations of amino acid residues of Ca^2+^ bowl and RCK domain, it appears in D994A and D433A mutant channels that the degree of gintonin-mediated BK_Ca_ channel activation was more strongly attenuated than that of acetylcholine-mediated BK_Ca_ channel activation. These results imply that although both agents use the same signaling pathway for BK_Ca_ channel activation, D994 residue in Ca^2+^ bowl and D433 residue in RCK domain might play more important role in gintonin- rather than acetylcholine-mediated BK_Ca_ channel activation.

**Figure 8:**
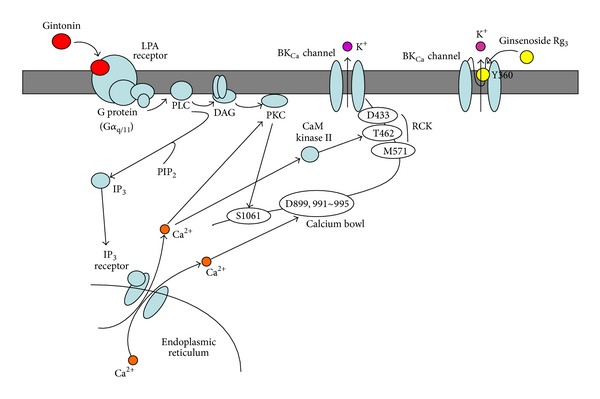
A comparative drawing of the action modes between gintonin and ginsenoside Rg_3_ in BK_Ca_ channel activation. Gintonin activates BK_Ca_ channels via G protein-coupled LPA1 receptors. Gintonin-mediated BK_Ca_ channel activations are mediated by Ca^2+^ binding to the Ca^2+^ bowl, RCK, or via activations of Ca^2+^-dependent kinases, whereas ginsenoside Rg_3_ activates BK_Ca_ channels through direct interaction with a specific amino acid located at the pore entryway of channel proteins following depolarization but not receptor activation [24].

In a previous study, we demonstrated that ginsenoside Rg_3_ enhances BK_Ca_ channel currents following depolarization [24]. By comparing the regulatory mode of gintonin action with ginsenoside Rg_3_ action for BK_Ca_ channel activation, we determined that gintonin differs from ginsenoside Rg_3_. Ginsenoside Rg_3_-induced enhancement of BK_Ca_ channel currents was not achieved through receptor-mediated transient [Ca^2+^]_i_ (Figures 2 and 3). Thus, ginsenoside Rg_3_-induced BK_Ca_ channel current enhancement did not include membrane receptor signaling transduction pathways. Instead, as a kind of dammarane glycosides (Figure 1(b)), ginsenoside Rg_3_-induced enhancement of BK_Ca_ channel currents was abolished by substitution of a Tyr360 residue, located at the channel pore entrance, and the enhancement of BK_Ca_ channel currents by Rg_3_ did not show desensitization after repeated treatment [24]. Thus, ginsenoside Rg_3_ regulates BK_Ca_ channel activity through direct interaction with channel proteins at the channel pore entrance. In contrast, as a G protein-coupled LPA receptor ligand, gintonin amplifies BK_Ca_ channel activation via a series of signal transductions through membrane bound G protein-coupled LPA receptor activation (Figure 8). Supporting this notion, gintonin, even at much lower concentrations than ginsenoside Rg_3_, induces greater amplitudes of outward BK_Ca_ channel currents (by 4-5-fold) than ginsenoside Rg_3_ (Figure 3). The EC_50_ of gintonin is about 35 nM (under the assumption that the molecular weight of gintonin is 20 kDa), whereas that of ginsenoside Rg_3_ was about 15 *μ*M for BK_Ca_ channel activation [24]. In addition, interruptions of the receptor signaling pathway by inhibitors or mutations abolished or attenuated gintonin-mediated but not ginsenoside Rg_3_-mediated BK_Ca_ channel activation. These results indicate that although ginseng contains two agents with two different action modes for the regulation of BK_Ca_ channel activity, gintonin is more efficient for BK_Ca_ channel activation than ginsenoside Rg_3_ (Figure 8).

BK_Ca_ channels are widely distributed in nervous and vascular systems [10, 45, 46]. *In vitro* gintonin-mediated BK_Ca_ channel activation might be associated with the *in vivo* pharmacological effects of ginseng. In previous studies, ginseng has exhibited neuroprotective effects against a variety of excitatory neurotransmitters, toxins, or ischemic stroke [1]. In addition, ginseng is also reported to induce relaxation of blood vessels constricted by adrenergic receptor stimulations [47, 48]. Thus, gintonin might be utilized for the reduction of overexcitability of the nervous system or to downregulate hyperactivity of blood vessels. Thus, the present studies show the possibility using a *Xenopus* oocyte gene expression model system that gintonin might participate in the regulation of synaptic transmission in nerve terminals and vascular muscle tone. However, more investigations are needed to extend from *Xenopus *oocytes to neuron or muscle cells.

In summary, we found that gintonin induces BK_Ca_ channel activation via membrane G protein-coupled LPA receptor signaling pathways. Using site-directed mutagenesis, we further confirmed the molecular mechanisms between the Ca^2+^ bowl, RCK domain, and CaM kinase II, which are involved in gintonin-mediated BK_Ca_ channel regulation. These novel findings provide insight into the molecular basis of the pharmacological effects of ginseng in the nervous and vascular systems.
